# Tau deposition is associated with functional isolation of the hippocampus in aging

**DOI:** 10.1038/s41467-019-12921-z

**Published:** 2019-10-25

**Authors:** Theresa M. Harrison, Anne Maass, Jenna N. Adams, Richard Du, Suzanne L. Baker, William J. Jagust

**Affiliations:** 1 Helen Wills Neuroscience Institute, UC Berkeley, Berkeley, CA USA; 2 German Center for Neurodegenerative Diseases, Magdeburg, Germany; 3 Lawrence Berkeley National Laboratory, Berkeley, CA USA

**Keywords:** Protein aggregation, Cognitive ageing, Learning and memory, Alzheimer's disease

## Abstract

The tau protein aggregates in aging and Alzheimer disease and may lead to memory loss through disruption of medial temporal lobe (MTL)-dependent memory systems. Here, we investigated tau-mediated mechanisms of hippocampal dysfunction that underlie the expression of episodic memory decline using fMRI measures of hippocampal local coherence (regional homogeneity; ReHo), distant functional connectivity and tau-PET. We show that age and tau pathology are related to higher hippocampal ReHo. Functional disconnection between the hippocampus and other components of the MTL memory system, particularly an anterior-temporal network specialized for object memory, is also associated with higher hippocampal ReHo and greater tau burden in anterior-temporal regions. These associations are not observed in the posteromedial network, specialized for context/spatial information. Higher hippocampal ReHo predicts worse memory performance. These findings suggest that tau pathology plays a role in disconnecting the hippocampus from specific MTL memory systems leading to increased local coherence and memory decline.

## Abstract (web summary)

Deposition of tau protein aggregates occurs during aging and Alzheimer disease. Here, the authors show that tau burden in the anterior-temporal memory network is associated with disrupted fMRI connectivity and functional isolation of the hippocampus from other memory network components.

## Introduction

Structural and functional alterations of the hippocampus are critical consequences of both normal aging and Alzheimer disease (AD)^1–5^. An underlying pathological process in both conditions is the accumulation of the microtubule-associated protein tau as neurofibrillary tangles (NFTs) in medial temporal lobe (MTL) structures that play an important role in episodic memory function^6^. It has long been suspected that tau pathology deprives the hippocampus of its main input by interfering with the origins of the perforant pathway in the entorhinal cortex (ERC), functionally isolating the structure^7^. Current imaging approaches now allow us to assess this hypothesis in living people.

The ERC has posteromedial and anterolateral subregions with unique patterns of connectivity and unique roles in memory function^8^. One model of normal MTL function emphasizes the hippocampus’ role in integrating item (anterolateral ERC—perirhinal cortex) and context (posteromedial ERC—parahippocampal cortex (PHC)) representations in episodic memories^9,10^. To do this, two distinct networks converge in the hippocampus: the anterior-temporal (AT) item or object network and the posterior medial (PM) context or spatial network^11^. In aging and AD, the regions comprising the AT (amygdala and areas of temporal cortex) and PM (PHC and posteromedial parietal cortex) networks are differentially targeted by early tau and β-amyloid (Aβ) pathology, respectively^12^.

Postmortem and longitudinal in vivo positron emission tomography (PET) data indicate that tau and Aβ accumulate in different spatial patterns and likely via different mechanisms^6,13–15^. Early Aβ deposition occurs across much of the cortex even in clinically normal aging (e.g., preclinical AD), while tau pathology begins in the transentorhinal cortex in the MTL and may spread in an activity-dependent manner along vulnerable functional networks^16–19^. Considerable evidence suggests that both pathological processes affect local brain activity and large scale neural networks, which in turn may affect cognitive function^12,20–23^. The relationships between Aβ, tau, connectivity in specific neural systems, and behavior may hold the key to better understanding of how early AD evolves in the aging brain.

**Fig. 1:**
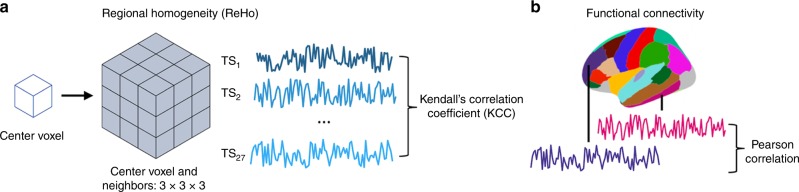
Local and distant functional connectivity can be measured using different, related approaches. **a** Regional homogeneity (ReHo) measures the nonparametric concordance of adjacent voxel timeseries (TS). For each voxel in the gray matter (center voxel in the figure), a Kendall’s correlation coefficient value is computed between that voxel’s TS and its 26 neighbors who share at least a face, edge or corner with the center voxel. ReHo is a measure of local connectivity. **b** Conventional functional connectivity measures statistical dependency, often Pearson correlation, between the time-varying fMRI BOLD signal in two voxels or regions. In this example, mean timeseries from two distant regions in a parcellation are correlated to estimate functional connectivity

Conventional functional connectivity (FC) uses fMRI to calculate statistical dependency, such as a Pearson’s correlation coefficient, between timeseries of spatially remote voxels or regions of interest (ROIs). Spatiotemporal correlation (autocorrelation) inherent to fMRI data makes it challenging to estimate the conventional FC of adjacent voxels or regions^24^. Instead, short-range, local connectivity can be measured using an approach called regional homogeneity (ReHo)^25^. ReHo is similar in principle to conventional FC, but explicitly measures nonparametric concordance of neighboring voxels so that patterns of local connectivity can be examined across the cortex (Fig. 1). Here, we use ReHo to measure local connectivity and conventional FC for longer-range connectivity to determine unique relationships of local and distant connectivity to AD pathology and cognition.

The goal of this study is to understand the mechanisms in aging and AD that affect hippocampal function and underlie the expression of episodic memory decline, which is not only an early sign of AD but is also observed in normal aging. To do this, we measure local (ReHo) and distant FC of the hippocampus in cognitively healthy older adults (OA) and young adults (YA). After examining the effect of age on connectivity measures, we then use PET measures of brain Aβ and tau deposition to determine how pathology affected local and distant connectivity of the hippocampus. We assess these relationships in ROIs selected a priori to represent the AT (tau-vulnerable ROIs) and PM (amyloid-vulnerable ROIs) memory networks because of their different vulnerability to tau and Aβ pathology and their known participation in MTL memory circuits. Finally, we relate hippocampal ReHo and FC to each other and to memory performance. Our underlying hypothesis is that tau deposition in the AT memory network will disconnect the hippocampus, increase local connectivity, and adversely affect memory function.

## Results

### Study participants

Eighty-nine cognitively healthy OA underwent resting state functional MRI (rs-fMRI) at 3 T, PET imaging and cognitive assessments. The mean age of the OA cohort was 77 ± 6.1(60–93) years and 63% were female (Table 1). All OA participants were community dwelling with normal cognitive performance and no medical illnesses or medications that affect cognition. Because a normative pattern of whole-brain ReHo is not well established, we also included 50 YA aged 20–35 years (mean age 25 ± 4.4 years, 50% female) to serve as reference group. YA underwent 3T rs-fMRI and completed cognitive testing, but did not receive PET scans because this age group is unlikely to have measurable AD pathology.

**Table 1:** Cohort characteristics

	YA (*n* = 50)	OA (*n* = 89)
Age	24.8 ± 4.4 (20–35)	77.0 ± 6.1 (60–93)
Sex (M/F)	25/25	33/56
Education (Yrs)	16.2 ± 2.0	16.7 ± 1.9
APOE ε4 (C/NC)	N/A	27/60 (2N/A)
Global PiB DVR	N/A	1.16 ± 0.24 (0.93–1.85) (2N/A)
PiB + /-	N/A	39/48 (2N/A)
PM PiB DVR	N/A	1.22 ± 0.23 (0.98–1.91) (2N/A)
AT PiB DVR	N/A	1.04 ± 0.14 (0.89–1.63) (2N/A)
ERC FTP SUVR	N/A	1.30 ± 0.25 (0.86–2.11)
AT FTP SUVR	N/A	1.28 ± 0.18 (0.95–2.02)
PM FTP SUVR	N/A	1.18 ± 0.12 (0.92–1.65)
Episodic memory (z-score)	0^a^ ± 0.641 (−1.66–1.06)	−0.02 ± 0.91 (−2.35–1.71)
Working memory (z-score)	0^a^ ± 1.000 (−1.64–1.90)	−0.24 ± 0.96 (−1.95–2.27)
Executive function (z-score)	0^a^ ± 0.602 (−1.28–1.60)	0.05 ± 0.73 (−1.61–2.39)

^a^Mean cognitive scores for YA are approaching zero because z-scores were created based on the mean and standard deviation of each test score in the same cohort

*YA* young adults, *OA* older adults, *M*  male, *F*  female, *Yrs*  years, *C*  carrier, *NC*  non-carrier, *N/A*  not available, *PiB*  Pittsburgh compound B, *DVR*  distribution volume ratio, *PM*  posteromedial, *AT*  anterior temporal, *ERC*  entorhinal cortex, *FTP*  flortaucipir, *SUV R* standardized uptake value ratio, *IT*  inferior temporal cortex

### Patterns of ReHo in gray matter change with age

**Fig. 2:**
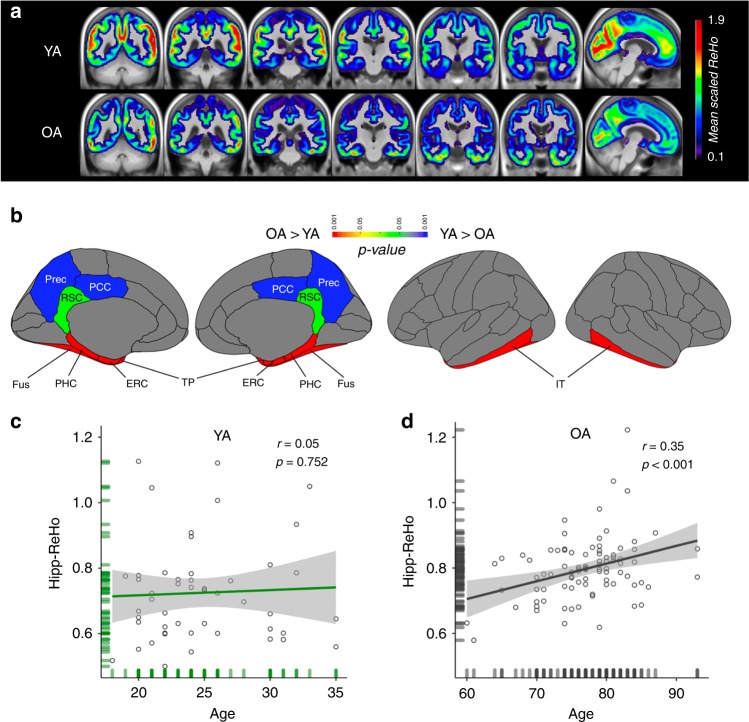
Patterns of ReHo in gray matter change with age. **a** Group mean ReHo images of YA (*n* = 50) and OA (*n* = 89) demonstrated distinct ReHo patterns. Areas of high ReHo in YA were lateral parietotemporal junction, precuneus, and primary visual areas. In OA, high ReHo was observed in a majority of the temporal lobe. **b** ANOVA was used to compare ReHo in a priori cortical ROIs between YA and OA. Differences in ReHo signal were observed in all illustrated ROIs except RSC where the difference was trending (*p* = 0.081). ReHo in AT ROIs, PHC, and ERC was significantly greater in OA than YA (all *p* < 0.001), while ReHo in PCC and Prec was significantly lower in OA than YA (all *p* < 0.001). **c** There was no relationship between age and hipp-ReHo in YA (*r* = 0.05, *p* = 0.75; all *r* and *p*-value pairs reflect Pearson correlation). **d** In contrast to YA, there was a significant positive relationship between age and hipp-ReHo in OA (*r* = 0.35, *p* < .001). Source data are provided as a Source Data file. YA young adults, OA older adults, Prec precuneus, PCC posterior cingulate cortex, RSC retrosplenial cortex, Fus fusiform, PHC parahippocampal cortex, ERC entorhinal cortex, TP temporal pole

For every participant, voxelwise ReHo was calculated within a participant-specific gray matter (GM) mask. ReHo values were scaled by the GM mean for each participant, warped to template space and averaged to create mean YA and OA ReHo images. In YA, areas of high ReHo included lateral parietotemporal junction, lateral frontal, precuneus, and primary visual areas with lower ReHo in temporal lobe (Fig. 2a). In contrast, high ReHo regions in OA included much of the temporal lobe. We also examined a set of a priori ROIs representing the AT (amygdala, fusiform (Fus), temporal pole (TP), inferior temporal gyrus (IT)) and PM (PHC, retrosplenial cortex (RSC), precuneus (Prec), posterior cingulate (PCC)) memory systems as well as areas of AT-PM system integration (ERC and hippocampus). OA had significantly higher ReHo than YA in areas associated with early tau pathology, including AT ROIs, ERC and hippocampus as well as the PM region PHC (amygdala: F(1, 137) = 4.15, *p* = 0.044; all other regions *p* < 0.001; Fus: F(1, 137) = 265; TP: F(1, 137) = 23.7; IT: F(1, 137) = 107; ERC: F(1, 137) = 150; hippocampus: F(1,137) = 11.8; PHC: F(1, 137) = 89.2; ANOVAs), and significantly lower ReHo in PCC and Prec (both *p* < 0.001; PCC: F(1, 137) = 13.3; Prec: F(1, 137) = 40.1; ANOVAs), regions associated with early Aβ pathology (Fig. 2b). OA ReHo was also lower than YA in RSC, an early Aβ region, but this difference was a trend (F(1, 137) = 3.1, *p* = 0.081; ANOVA). Differences in cortical ROIs remained significant when adjusting for ROI thickness, but differences in amygdala and hippocampus were not significant after adjusting for ROI volume. Given our focus on hippocampus, we examined the relationship between age and hipp-ReHo in each cohort. Within OA, there was a significant positive relationship between hipp-ReHo and age (*r* = 0.35, *p* < 0.001; all *r* and *p*-value pairs reflect Pearson correlation; Fig. 2d). There was no association between hipp-ReHo and age within YA (*r* = 0.05, *p* = 0.75; Fig. 2c).

A number of factors related to age-related changes in the BOLD signal or atrophy could artefactually drive differences between YA and OA. We first investigated whether perfusion could be driving ReHo measurements by deriving a hippocampal cerebral blood flow estimate from the dynamic Aβ PET data (OA only) and found no association between perfusion to the hippocampus and hipp-ReHo (*r* = −0.02, *p* = 0.83; Supplementary Fig. 1). We next investigated age-related variability (standard deviation)^26^ in mean hippocampal BOLD timeseries across both YA and OA and found no relationship with hipp-ReHo (Supplementary Notes and Supplementary Fig. 2). Finally, we found a weak negative association between hippocampal volume and ReHo (*r* = −0.22, *p* = 0.03; Supplementary Fig. 1) that did not survive adjustment for age and sex (*p* = 0.59; multiple regression). Nevertheless, subsequent analyses adjusted for age, sex, and hippocampal volume to ensure we were detecting relationships statistically independent from the effects of these covariates (figures depict raw, non-residualized data).

### Hipp-ReHo is associated with AD pathology in older adults

**Fig. 3:**
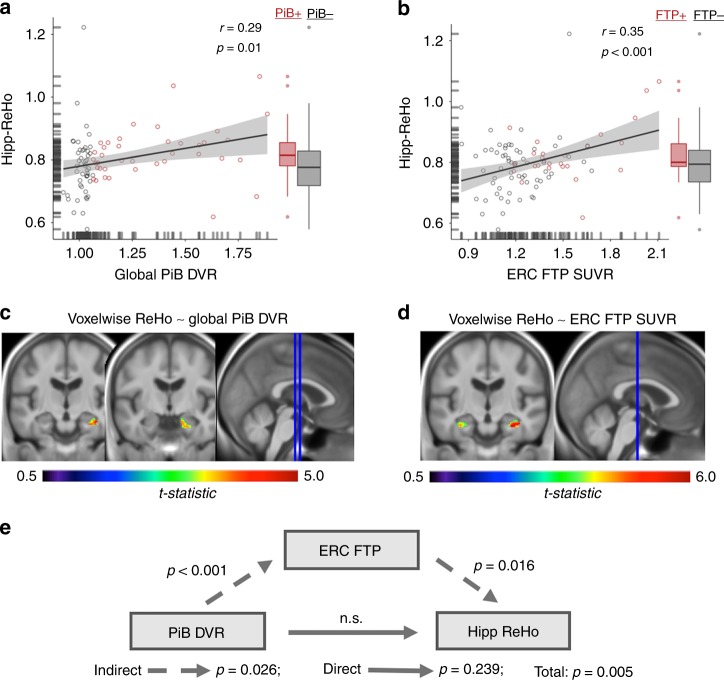
Hipp-ReHo is associated with AD pathology in older adults. **a** Global PiB DVR (PiB + in red; PiB- in gray; threshold DVR > 1.065) positively predicted hipp-ReHo (*r* = 0.29, *p* = 0.01; all *r* and *p*-value pairs reflect Pearson correlation). Global PiB DVR remained a significant predictor of hipp-ReHo after adjusting for age, sex, and hippocampal volume. The band within the boxplots represents the median while the upper and lower edges of the box represent the first and third quartiles, respectively. The whiskers extend up to 1.5 times the interquartile range. Data points outside this range are plotted as outliers. **b** ERC FTP SUVR (FTP + in red; FTP- in gray; threshold SUVR > 1.26 in Braak III/IV) was a positive predictor of hipp-ReHo (*r* = 0.35, *p* < 0.001). ERC FTP SUVR remained a significant predictor of hipp-ReHo after adjusting for age, sex and hippocampal volume. Illustrative boxplots are used as in panel A. **c**, **d** Voxelwise regressions of ReHo images spanning all gray matter voxels with global PiB DVR or ERC FTP SUVR as the independent variable of interest illustrate significant clusters (cluster corrected *p* < 0.05) predominantly within the hippocampus. **e** A mediation analysis testing if ERC FTP SUVR mediates the observed relationship between PiB DVR and hipp-ReHo revealed a significant mediation effect (indirect path *p* = 0.025; direct path *p* = 0.239; total *p* = 0.005). Source data are provided as a Source Data file. ReHo regional homogeneity, PiB Pittsburg compound B, DVR distribution volume ratio, ERC entorhinal cortex, FTP flortaucipir, SUVR standardized uptake value ratio

Next, in OA, we examined the relationship between hipp-ReHo and Aβ and tau pathology measured with PET. Aβ was quantified using the Pittsburg compound B (PiB) tracer and tau was measured using flortaucipir (FTP). For PiB, dynamic data were available and distribution volume ratio (DVR) was calculated for a large cortical ROI to obtain a global PiB DVR, a standard measure of total Aβ burden. We also measured mean PiB DVR in the Aβ-vulnerable PM network as a single ROI. Global PiB DVR and PM PiB DVR were highly correlated (*r* = 0.99, *p* < 0.001). Using global PiB DVR or PM PiB DVR, we found a significant positive relationship between Aβ burden and hipp-ReHo (global: *r* = 0.29, *p* = 0.01, Fig. 3a; PM: *r* = 0.27, *p* = 0.01, Supplementary Table 1). The associations between Aβ burden and hipp-ReHo remained significant after adjusting for age, sex and hippocampal volume (*p*’s < 0.02; multiple regression). OA *APOE* ε4 carriers (*n* = 27 [all heterozygotes, 2 ε2/ε4] vs. 60 non-carriers; 2 genotypes NA) had significantly greater Aβ burden (*p* < 0.001; t-test), but *APOE* was not related to hipp-ReHo (*p* = 0.134; t-test). A mean voxelwise PiB map showed a broad distribution of tracer uptake across much of cortex, as expected (Supplementary Fig. 3).

For FTP, standard uptake value ratio (SUVR) was calculated from data obtained at 80–100 min post-injection. SUVRs for Freesurfer-defined ERC, IT and the tau-vulnerable AT network were calculated after partial volume correction^27^. We chose to examine ERC because it is the site of earliest cortical tau accumulation and IT because is one of the earliest sites of extra-MTL tau spread and shows a large effect size distinguishing impaired from unimpaired individuals^28,29^. IT is also included in the larger AT network ROI, but we chose to also examine IT separately given previous work identifying this as a key ROI for measuring FTP signal. FTP measures in ERC, IT and AT were correlated with each other (*r*’s = 0.66–0.89, *p* < 0.001), but to a lesser degree than PiB measures. As with PiB, there was a positive correlation between FTP signal in ERC and hipp-ReHo (*r* = 0.35, *p* < 0.001; Fig. 3b). The relationship between hipp-ReHo and FTP signal was also present in AT regions (*r* = 0.37, *p* < 0.001) but not PM regions (*r* = 0.15, *p* = 0.156; Supplementary Table 1). Associations between tau pathology and hipp-ReHo remained significant when adjusting for age, sex, and hippocampal volume (ERC *p* = 0.008; IT *p* = 0.004; AT *p* = 0.002; multiple regression). We found no significant relationship between pathology and ReHo measured in other a priori ROIs (*p*’s > 0.14). A mean voxelwise FTP map showed a temporal-predominant pattern of tracer uptake, as expected (Supplementary Fig. 3).

To examine whether an ROI-based approach might have missed associations between Aβ or tau pathology and ReHo, we warped voxelwise ReHo images to standard space and ran exploratory voxelwise regressions across the entire gray matter using global PiB DVR or ERC FTP SUVR as the independent variable of interest. In both regressions, the only significant clusters that were observed were predominantly in the hippocampus (Fig. 3c, d). In Fig. 3c the cluster falls at the transition zone between hippocampus and adjacent perirhinal cortex or PHC.

It is well established that in AD Aβ and tau are correlated and that Aβ pathology likely precedes extensive neocortical tau accumulation^30–33^. Thus, we ran a mediation analysis to determine if the relationship we observed between global PiB DVR and hipp-ReHo was mediated by ERC FTP SUVR. Indeed, the model indicated that FTP signal in ERC significantly mediates the effect of global PiB signal on hipp-ReHo (indirect *p* = 0.026; direct *p* = 0.239; total *p* = 0.005; Fig. 3e). Results were similar if PM Aβ and AT tau were used as the predictor and mediator, respectively (indirect *p* = 0.024; direct *p* = 0.267; total *p* = 0.005). We did not find evidence that global PiB DVR mediated the relationship between ERC FTP SUVR and hipp-ReHo (indirect path *p* = 0.25; direct path *p* = 0.02; total *p* < 0.001).

### Hipp-ReHo is related to hippocampal functional isolation

**Fig. 4:**
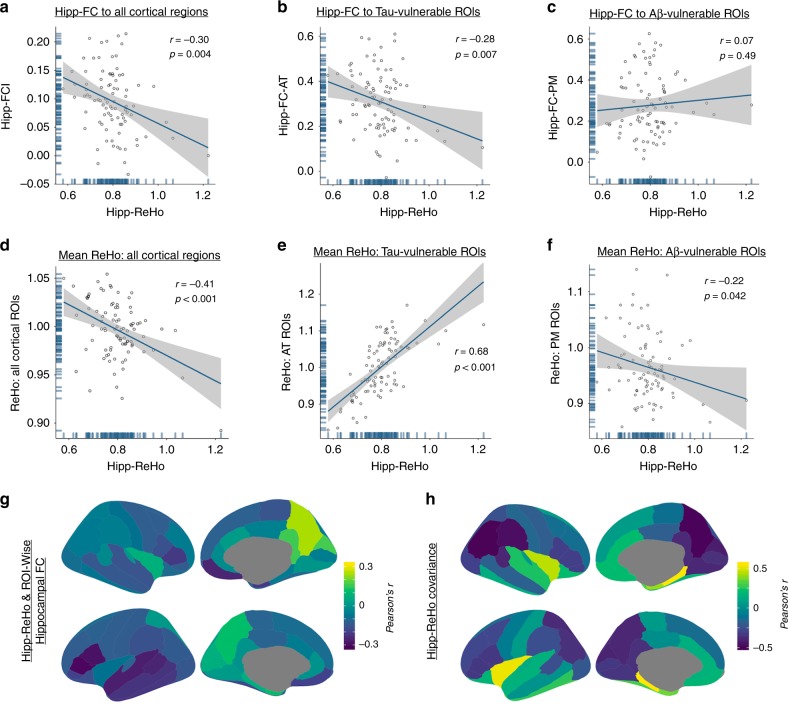
Higher hipp-ReHo is associated with functional isolation of the hippocampus. **a** Hipp-ReHo and hipp-FCI, a measure of hippocampal connectivity averaged over the entire cortex, were negatively correlated (*r* = −0.30, *p* = 0.004; all *r* and *p*-value pairs reflect Pearson correlation). **b** Hipp-ReHo was negatively correlated to hipp-FC-AT, a measure of hippocampal connectivity to AT regions (*r* = −0.28, *p* = 0.007). **c** There was no relationship between hipp-ReHo and hipp-FC-PM (*r* = 0.07, *p* = 0.49). **d** Hipp-ReHo negatively predicted mean ReHo across all cortical ROIs (*r* = −0.41, *p* < 0.001). **e** Hipp-ReHo was positively correlated to mean ReHo in AT regions (*r* = 0.68, *p* < 0.001). **f** Hipp-ReHo negatively predicted mean ReHo in PM regions (*r* = −0.22, *p* = 0.042). **g** Across OA participants, the correlation between hipp-ReHo and hippocampal FC to each cortical ROI indicated that increasing hipp-ReHo was related to decreasing FC between hippocampus and temporal cortex. **h** Across OA participants, hipp-ReHo covariance to ReHo in each cortical ROI showed that higher hipp-ReHo was associated with increased ReHo in temporal cortex. Source data are provided as a Source Data file. hipp-FCI hippocampal functional connectivity index, hipp-ReHo hippocampal ReHo, Hipp-FC hippocampal functional connectivity, AT anterior temporal, PM posterior medial, hipp-FC-AT hippocampal FC to tau-vulnerable AT regions, hipp-FC-PM hippocampal FC to Aβ-vulnerable PM regions

The relationship between higher hipp-ReHo and greater tau pathology in early accumulating regions that form a hippocampal-temporal lobe memory network raises the question of whether hipp-ReHo is related to FC between the hippocampus and other network components. First, we used conventional FC measures to examine a hippocampal FC index (hipp-FCI) which measured the average FC between the hippocampus and all Freesurfer-derived cortical ROIs. FC was calculated ROI-to-ROI using Fisher’s z-transformed Pearson correlations between mean timeseries. We observed a significant negative correlation between hipp-FCI and hipp-ReHo (*r* = −0.30, *p* = 0.004; Fig. 4a), indicating that as hipp-ReHo increased, FC between the hippocampus and cortex decreased. Next, we examined average hippocampal FC to the a priori tau-vulnerable AT ROIs (hipp-FC-AT) and to Aβ-vulnerable PM ROIs (hipp-FC-PM). Similar to the hipp-FCI results, higher hipp-ReHo predicted lower hipp-FC-AT (*r* = −0.28, *p* = 0.007; Fig. 4b). There was no significant relationship between hipp-ReHo and hipp-FC-PM (*r* = 0.07, *p* = 0.49; Fig. 4c). In YA, these relationships were not observed (Supplementary Fig. 2). In OA, we next visualized whole-brain spatial patterns of hipp-ReHo and FC associations by plotting the strength of the correlation across participants between hipp-ReHo and hippocampal FC to each cortical ROI (Fig. 4g). This demonstrates the negative correlations between hipp-ReHo and hippocampal FC with temporal lobe regions, and also shows similar negative associations between hipp-ReHo and hippocampal FC to the inferior frontal gyrus and medial prefrontal cortex. There was a positive association between hipp-ReHo and hippocampal FC to right precuneus (*r* = 0.24, *p* = 0.02).

To better understand how ReHo in the hippocampus affected the local connectivity of other areas of the brain, we examined how hipp-ReHo covaried with ReHo across other cortical regions. With an approach complimentary to the FC analyses, we correlated hipp-ReHo to (1) mean ReHo across all cortical ROIs, (2) mean ReHo in tau-vulnerable AT regions, and (3) in Aβ-vulnerable PM regions. Hipp-ReHo was negatively associated with average ReHo across the cortex (*r* = −0.41, *p* < 0.001; Fig. 4d). In contrast, there was a strong positive correlation between hipp-ReHo and ReHo in AT regions (*r* = 0.68, *p* < 0.001; Fig. 4e). There was a negative correlation between hipp-ReHo and ReHo in PM regions (*r* = −0.22, *p* = 0.042; Fig. 4f). For visualization and comparison to the FC results, we plotted the strength of the correlation across participants between hipp-ReHo and ReHo in each cortical ROI (Fig. 4h). We observed that temporal regions showed increases in ReHo which positively covaried with hipp-ReHo. The whole-brain hipp-ReHo covariance also revealed positive correlations with regions outside the temporal lobe, such as orbitofrontal cortex and inferomedial frontal cortex.

### Hippocampal FC is related to tau outside the MTL

**Fig. 5:**
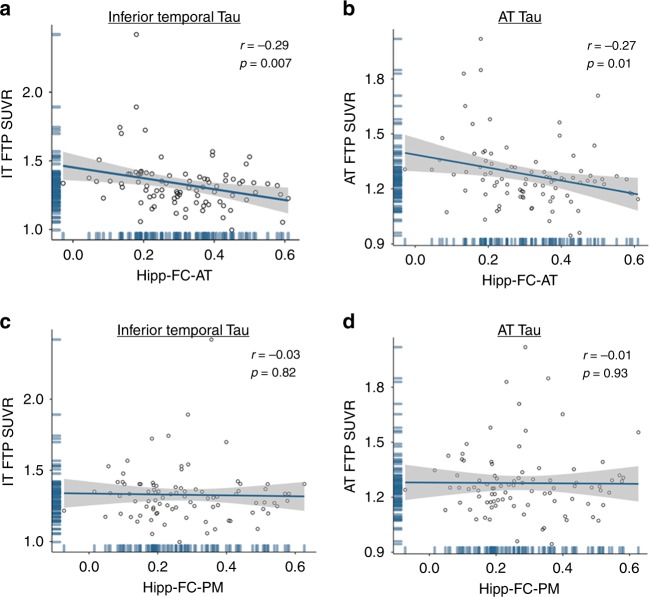
Hippocampal FC is related to tau outside the MTL. **a** Hipp-FC-AT, measure of hippocampal connectivity to AT regions, negatively predicted IT FTP SUVR (*r* = −0.29, *p* = 0.007; all *r* and *p*-value pairs reflect Pearson correlation;). Hipp-FC-AT remained a significant predictor of FTP SUVR in IT (*p* = 0.04; multiple regression) after adjusting for age, sex, hippocampal volume and hipp-ReHo. **b** Hipp-FC-AT was a negative predictor of AT Tau SUVR (*r* = −0.27, *p* = 0.01). After adjusting for covariates, hipp-FC-AT predicted AT FTP SUVR at statistical trend level (*p* = 0.06; multiple regression). **c**, **d** There was no relationship between Hipp-FC-PM and IT FTP SUVR (*r* = −0.03, *p* = 0.82) or AT FTP SUVR (*r* = −0.01, *p* = 0.93). Source data are provided as a Source Data file. IT inferior temporal cortex, FTP flortaucipir, SUVR standardized uptake value ratio, AT anterior temporal, PM posterior medial, hipp-FC-AT hippocampal functional connectivity to tau-vulnerable AT regions, hipp-FC-PM hippocampal functional connectivity to Aβ-vulnerable PM regions

To discover if hippocampal FC, like hipp-ReHo, was related to pathology, we tested whether hipp-FCI, hipp-FC-AT or hipp-FC-PM was associated with FTP signal. Without adjustment for covariates, lower hipp-FCI was related to greater FTP signal in ERC (*r* = −0.33, *p* = 0.002), IT (*r* = −0.31, *p* = 0.003) and AT (*r* = −0.28, *p* = 0.009). Results with hipp-FC-AT were similar, showing a negative association with FTP signal in ERC (*r* = −0.22, *p* = 0.047), IT (*r* = −0.29, *p* = 0.007; Fig. 5a) and AT (*r* = −0.27, *p* = 0.011; Fig. 5b). However, after adjusting for age, sex, hippocampal volume, and hipp-ReHo, only the association between hipp-FC-AT and FTP signal in IT (*p* = 0.04; multiple regression) remained significant, while the hipp-FC-AT relationship with AT FTP was reduced to a trend (*p* = 0.06). Hipp-FC-PM was not related to tau pathology (*p*’s > 0.322; Pearson correlation; Fig. 5c, d). Thus, only hippocampal FC to tau-vulnerable AT regions was robustly related to tau measures, specifically in regions outside the MTL like IT. Aβ burden across the cortex or in PM regions was not related to FC measures (all *p*’s > 0.2; Pearson correlation).

### Hipp-ReHo predicts episodic memory

**Fig. 6:**
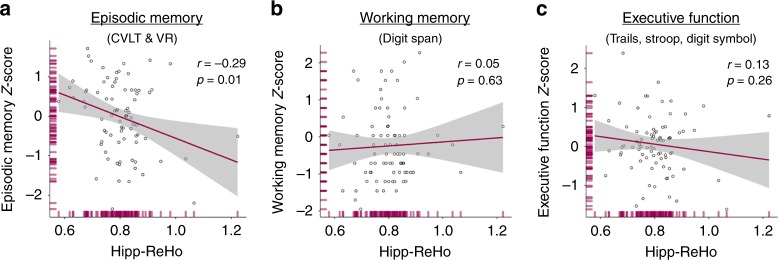
Hipp-ReHo predicts episodic memory but not working memory or executive function. **a** Higher hipp-ReHo predicted worse episodic memory performance (*r* = −0.29, *p* = 0.01; all *r* and *p*-value pairs reflect Pearson correlation;). In a multiple regression, hipp-ReHo remained a significant predictor of memory (*p* = 0.046) after adjusting for age, sex, hippocampal volume and global PiB DVR, but was trending (*p* = 0.094) in a model that included ERC FTP SUVR, which was also trending (*p* = 0.070). **b**, **c** There was no significant relationship between hipp-ReHo and working memory (*r* = 0.05, *p* = 0.63) or executive function (*r* = −0.13, *p* = 026). Source data are provided as a Source Data file. ReHo regional homogeneity, hipp-ReHo hippocampal ReHo, PiB Pittsburgh compound B, DVR distribution volume ratio

Cognition was assessed as a mean z-score of standard neuropsychological tests in three different domains: episodic memory, working memory, and executive function. With evidence that higher hipp-ReHo is associated with tau pathology and reduced FC of the hippocampus, we hypothesized that hipp-ReHo would predict memory performance. Indeed we found that higher hipp-ReHo predicted worse episodic memory performance (*r* = −0.29, *p* = 0.01; Fig. 6a), and that this relationship remains significant when adjusting for age, sex, hippocampal volume, and global PiB DVR (*p* = 0.046; multiple regression). When ERC FTP SUVR was added to this multiple regression model instead of global PiB DVR, only hippocampal volume remained a significant predictor of episodic memory but hipp-ReHo (*p* = 0.094) and ERC FTP SUVR (*p* = 0.070) were both trending (Supplementary Table 2). In contrast, hipp-ReHo was not related to working memory or executive function (p’s > 0.26; Pearson correlations; Fig. 6b, c). Furthermore, memory was not related to mean ReHo in any of the other a priori ROIs (*p*’s > 0.127; Pearson correlations). According to these findings, the relationship between hipp-ReHo and cognition is specific to memory and the relationship between ReHo and memory is specific to the hippocampus. Finally, while we observed a relationship between hipp-ReHo and episodic memory performance, there was no association between hippocampal FC measures and memory (*p*’s > 0.381; Pearson correlations). In YA, there was no observed relationship between hipp-ReHo and episodic memory domain score (*r* = 0.05, *p* = 0.74) but ceiling effects may make an association difficult to observe (Supplementary Notes).

## Discussion

Our findings in cognitively normal older adults indicate that local connectivity of the hippocampus, measured with ReHo, was increased with age and tau pathology in ERC and the object/AT memory network. Hipp-ReHo was also increased with greater hippocampal disconnection from cortex and especially the AT network. Tau deposition in the AT network, in turn, was associated with functional disconnection of the hippocampus, measured with hipp-FC to AT regions. Higher hipp-ReHo was also related to advancing age and lower hippocampal volume, supporting the interpretation that greater local connectivity in the hippocampus is a malignant process. Finally, greater local connectivity in the hippocampus was associated with worse episodic memory function. Although the associations revealed by these data are cross sectional, the most likely interpretation, based on neuropathological evidence and neural system models of memory, is that tau deposition drives hippocampal disconnection, leading to increased hippocampal local connectivity and episodic memory decline.

We interpreted our findings based on a model that includes both age and AD pathology-related effects. We found that age was associated with changes in cortical ReHo patterns, including dramatic increases in temporal ReHo. Following on this observation of an age effect in temporal lobe, we explored the effect of local tau pathology on ReHo and found a robust relationship between hipp-ReHo and tau in each ROI we explored. Our interpretation of the relationship between tau and hipp-ReHo was that tau accumulation, especially in ERC, has the effect of disconnecting the hippocampus from its functional partners resulting in increased local synchrony within the hippocampus. To test this idea, we measured hipp-FC to whole cortex, PM and AT regions and related these measures to hipp-ReHo. We found that as hipp-ReHo increased,  hippocampal connectivity to whole cortex and AT regions decreased. While the resolution of our fMRI data prohibits assessment of hippocampal-ERC connectivity, the disruption of hippocampal connectivity to both diffuse cortical and AT regions may reflect the loss of inputs to the hippocampus due to tau accumulation in both ERC and AT brain regions. Further, reduced hippocampal connectivity to the AT network was associated with tau pathology in AT regions.

The pathological, anatomic and network relationships of these effects are compelling and specific. For example, hipp-ReHo was elevated in relation to tau in ERC and the AT network, and Aβ effects are mediated through tau. The increase in hipp-ReHo was related to functional disconnection between hippocampus and the AT network, but not to hippocampal disconnection from the PM network. Hippocampal connectivity to AT was, in turn, negatively related to tau deposition in the AT network, particularly in inferior temporal cortex. The known patterns of pathology and hippocampal connectivity informs the interpretation of these results. Layer II of ERC is severely affected by tau NFT pathology^7^; these neurons give rise to the perforant pathway relaying cortical information to the hippocampus, including dentate gyrus and CA fields^34^. Tau pathology in ERC affects distinct memory networks as lateral and medial subregions of ERC serve as relays for AT and PM networks, respectively^35^. Consistent with preponderant AT associations with early tau pathology, the lateral aspect of the ERC is involved first in aging and AD^6,36–38^. Our data suggest that age-related accumulation of tau, followed by spreading of tau potentiated by Aβ, disrupts functional memory circuits by disconnecting key MTL structures, like the hippocampus. This leads to increased local connectivity within the hippocampus, perhaps via disinhibited trisynaptic loop activity, which is associated with worse memory performance. While not addressable in the current study, it is important to note there may be other cellular mechanisms driving functional changes in the hippocampus, including reduced inhibitory cholinergic input to CA3 (ref.^39^).

Because our interpretation is based on cross-sectional observations, it is reasonable to question whether causal associations may be the opposite of what we propose. It is possible, for example, that hippocampal connectivity changes drive tau accumulation. While there is evidence in humans of an association between tau spread and the integrity of MTL neuroanatomical connections^40^, the direction of this association is unknown. Because current theories posit that tau spreads via activity-dependent release and transfer through neural connections, it seems that tau deposition would necessarily precede disruptions of connectivity (i.e., spread requires connectivity)^16,41,42^. Similarly, the possibility that increased hippocampal local connectivity drives functional disconnection seems less likely in view of the neuropathology data summarized above, indicating a major role for tau-related disconnection. Our interpretation is also is in line with electrophysiological work in mice that has shown that Aβ is associated with hyperactivation while tau is associated with reduced activity and neuronal silencing^43^. The predominant model of AD pathogenesis describes Aβ accumulation driving tau spread, which then has more proximal detrimental effects on neuronal function and behavior^44^. We believe our findings support this model and extend it by examining multiple connectivity measures and uncovering how disconnection in the presence of tau leads to local connectivity changes which are, in turn, predictive of behavior.

The findings of relationships between tau pathology, memory networks, and episodic memory dysfunction are remarkable for their detection in cognitively healthy OA. This suggests that local and distant connectivity changes in the hippocampus could be among the earliest functional changes in preclinical AD. We observed dramatic changes in cortical ReHo patterns in older adults compared to young adults, but associations between memory, hippocampal local coherence and distant connectivity largely were not observed in the young adult cohort stressing the role of both aging and AD pathology in these associations. Previously, a single study examined the effect of Aβ positivity on ReHo across the cortex in older adults, and reported results in line with ours, specifically identifying higher ReHo in fusiform and lower ReHo in precuneus in individuals with higher Aβ burden^45^. Based on our findings, it is possible that these results were driven by tau pathology which was not available in the cohort. Other studies in humans with MCI or AD have reported increased ReHo, and animal models of AD have reported associations between ReHo and tau deposition^46–48^.

Identifying the functional correlates of early deposition of AD pathology will be a key component in linking the emergence of Aβ and tau with cognitive decline and, eventually, clinical disease. Here we describe a positive relationship between tau pathology and local connectivity in the hippocampus, which is also related to worse memory performance on neuropsychological tests. Previous work has reported hyperactivity of the hippocampus during memory tasks related to aging, the presence of Aβ and a diagnosis of MCI^5,22,49–51^. In these studies, however, tau was not measured which makes it difficult to disentangle possible effects of tau accumulation on hippocampal activation. More recent work where tau burden was assessed has provided evidence that tau may be driving abnormal hippocampal activation^12,20,23^. Tau pathology in older adults is related to memory while Aβ burden is generally not predictive of cross-sectional cognition, and a similar pattern may be emerging as we examine the effect of AD pathology on fMRI measures such that tau is more proximal to functional changes that affect behavior^29,52,53^.

One of the key findings in the present study is that increased local connectivity in the hippocampus may signal increasing functional isolation of the structure. While the directionality of this association may be questioned, we observed overlapping patterns of where hipp-ReHo positively covaries with regional ReHo and where hipp-ReHo and regional hippocampal connectivity were negatively correlated. The overlap was most notable in the temporal lobe, but also included orbitofrontal cortex, a connected region downstream from the AT network components we examined (Fig. 4g, h)^11^. Our results show that as hipp-ReHo increases (worsens) ReHo also increases in the AT network, which is perhaps related to hippocampal disconnection (lower hipp-FC) from these regions. Interestingly, in covariance analyses hipp-ReHo was positively associated with ReHo in the AT network, but negatively associated with ReHo across the cortex and in the PM network (Fig. 4d–f). Thus, it may be that disconnection in the presence of tau leads to increases in local connectivity, while regions less affected by early tau pathology (e.g., PM regions) show decreases in ReHo. Further work is needed to better understand the drivers of decreasing ReHo in posteromedial cortical regions, which we also observed in OA compared to YA (Fig. 2a, b). The overall pattern is consistent with the idea that hippocampus becomes functionally isolated in aging and AD and indicates that this degradation of normal inputs, in the presence of tau pathology, is also reflected in local connectivity changes within the hippocampus itself.

MTL disconnection has been previously reported in the literature, and several studies have examined the MTL local connectivity environment in addition to distant connectivity. In the aging literature, the disconnection of hippocampus from neocortical regions has been reported and was associated with increased connectivity within MTL regions as well as episodic memory performance^54,55^. In the AD literature, early reports showed that the MTL, often reported as a node in the default mode network (DMN), becomes disconnected from the DMN as AD advances^4,56,57^. This disconnection phenotype includes reduced connectivity between MTL and posteromedial regions, a key component of the DMN and also an early site of Aβ accumulation^58^. The consequences of hippocampal disconnection remain incompletely understood, but there is growing evidence that changes in distant connectivity of hippocampus come hand-in-hand with local connectivity changes, as we report in the present study using ReHo. Using another approach to measure local connectivity, Pasquini and colleagues reported progressively increased local hippocampal connectivity across the AD clinical spectrum^59^. This increased local connectivity was negatively correlated to their measure of “global” hippocampal connectivity, and hippocampal local connectivity predicted episodic memory performance. Using both ReHo and conventional functional connectivity, we have replicated these main findings in older adults and extended them by integrating measures of Aβ and tau pathology and demonstrating that tau is related to hipp-ReHo. Another study also found that in patients with MCI as MTL becomes less functionally connected with DMN, there is increased intra-MTL connectivity (e.g., between ERC and anterior hippocampus), indicating greater local connectivity in MTL^60^. There is also evidence of increased hippocampal glucose metabolism with decreased hippocampal functional connectivity^61^. Together, these findings using a variety of different approaches, indicate relationships between disconnection of the hippocampus and local connectivity and metabolism that strongly parallel our results.

We observed an association between increased local connectivity of the hippocampus and episodic memory performance in older adults (Fig. 6a). ReHo in other temporal regions was not related to memory. Further, hipp-ReHo was not related to working memory or executive function scores. Thus, the relationship between ReHo and cognition was specific to this association between hippocampus and memory. Our model posits that disconnection of the hippocampus and increased hipp-ReHo represent tau-associated dysfunction of this key memory structure. Thus, the relationship between hipp-ReHo and memory performance is a critical component of our findings because it demonstrates that there is a behavioral consequence of the functional changes we measured. Still, we acknowledge that episodic memory is a complex cognitive function and multiple parameters affect memory including age, tau pathology, hippocampal volume, and hipp-ReHo (Supplementary Table 2). We did not, however, find evidence of a relationship between hipp-FC measures and memory, suggesting that hipp-ReHo is a better measure of age and pathology-related hippocampal dysfunction than conventional functional connectivity.

This study has several limitations. First, the cross-sectional design limits causal inferences. Mediation analyses performed with cross-sectional data have been shown to not necessarily reflect mediation across time, which requires longitudinal data to establish^62^. Second, while the sample size of the study was reasonable, some relationships were strongly influenced by a few individuals. These individuals appear to be more impaired and, while cognitively normal, likely closer to the expression of disease symptoms. Their exclusion would limit the range of biomarker results in normal aging. Third, there is no gold standard distinction between local and distant connectivity. Local connectivity has previously been defined as regions or voxels ≤ 14 mm apart^63^. With our ReHo approach, each voxel neighborhood is a cube with 7.8 mm sides, well within the local connectivity distance window. However, some of the hippocampal connectivity we measured (e.g. hippocampus to PHC, a PM ROI), using standard approaches, had ROI centers of mass located less than 14 mm apart. We mitigated this by confirming FC results in regions that are remote and reported FC patterns within AT and PM memory networks as a whole. Finally, 3D ReHo inherently ignores individual differences in cortical folding and is prone to partial volume effects at the edges of tissue types^64^. We managed this problem by examining relationships between ReHo and volume, and including volume as a covariate. This did not substantially affect our results.

This study, focused on early hippocampal dysfunction in aging, integrates imaging across multiple modalities and incorporated the effect of AD pathology on connectivity. It will be important in the future to examine task fMRI data in cognitively healthy OA and mild MCI to determine if hipp-ReHo is associated with pathology-related aberrant hippocampal activation and with associative memory task performance. Ultimately hipp-ReHo, a conceptually simple resting state metric of clinically meaningful hippocampal functional change, could be useful in characterizing hippocampal function in impaired populations for which participation in a memory task paradigm would be challenging or unfeasible.

The processes that underlie cognitive decline in aging are poorly understood, but it is becoming increasingly apparent that the protein aggregates associated with AD—Aβ and tau—are often involved. Recent reports indicate relationships between tau accumulation and episodic memory decline in normal older people^29,53^. The deposition of tau in MTL brain regions which are intimately associated with memory function is not likely to be a coincidence in view of the well-established associations between tau, memory, aging, and AD. Here we used local and distant connectivity of the hippocampus to try to link pathology to the functional landscape of the aging hippocampus and, ultimately, cognition. With this approach, our work provides a mechanistic explanation of how tau may lead to hippocampal dysfunction. It also points towards a measurable fMRI variable, regional homogeneity, that could be useful as an early indicator of cognitive dysfunction.

## Methods

### Participants

The present study included 89 cognitively healthy older adult (OA) participants enrolled in the Berkeley Aging Cohort Study (BACS), an ongoing longitudinal study of normal cognitive aging. All participants were required to be age 60 or older with sMRI and rs-fMRI scans at 3T as well as PiB-PET and FTP-PET data quantifying Aβ and tau in the brain, respectively. We required that the 3T MRI and PET acquisition sessions were no more than 6 months apart. Additional eligibility requirements included that all participants were community dwelling, had a baseline MMSE score ≥ 25, normal performance on cognitive tests (within 1.5 SD of normative values on the California Verbal Learning Test and Delayed Recall from the Wechsler Memory Scale), no neurological, psychiatric or major medical illness and were taking no medications affecting cognition.

Using the same criteria (except age) we enrolled 50 young adults (YA) aged 20–35 to serve as a young reference group. Beside differing in age from the older adults, the YA participants also did not undergo PET data collection. The YA participants participated in neuropsychological testing (*n* = 44; 6 missing) as well as 3T sMRI and rs-fMRI scanning.

The Institutional Review Board at Lawrence Berkeley National Laboratory and the University of California Berkeley approved the present study and written, informed consent was obtained from all participants.

### Neuropsychological assessment

All participants in the BACS undergo neuropsychological testing to measure performance on specific cognitive tasks including those related to verbal and visual memory, working memory, processing speed, executive function, language, and attention. In the present study, composite scores were calculated to measure three cognitive domains: episodic memory, working memory, and executive functioning. Episodic memory tests were California Verbal Learning Test (CVLT) immediate and long delay free recall totals and the Visual Reproduction (VR) immediate and delay recall totals. Working memory was assessed by Digit Span total score. Processing speed tests were Trail Making Test B minus A, Stroop number correct in 1 min and Digit Symbol total. For episodic memory and executive function, domain scores were calculated by calculating the average z-score of the constituent tests. For OA, z-scores for a specific test were calculated using the mean and standard deviation computed from older BACS participants with neuropsychological data regardless of imaging requirements (up to one missing cognitive test allowed, *n* = 155). We chose to use this larger group as the z-score reference cohort instead of the study cohort for increased accuracy of the mean and variance estimates. For YA, z-scores were calculated based on the means and standard deviations of the cohort included in the present study.

### Image acquisition: MRI

MRI data at 3-Tesla (3 T) were collected at the Henry H. Wheeler Jr. Brain Imaging Center at UC Berkeley on a Siemens TIM/Trio scanner using a 32-channel head coil. First, a whole-brain T1-weighted magnetization prepared rapid gradient echo (MPRAGE) sMRI scan was acquired with the following parameters: sagittal slice orientation, 160 slices, repetition time (TR) = 2300 ms, echo time (TE) = 2.98 ms, matrix = 256 × 240, flip angle = 9°, voxel size = 1 mm isotropic. For each participant the sMRI was followed by a 5-min 20-s rs-fMRI run comprised of 300 T2*-weighted, whole-brain echo-planar images (EPIs) with a 2.6 mm isotropic voxel size and the following acquisition parameters: sagittal slice orientation, 60 slices, TR = 1067 ms, TE = 31.2 ms, matrix = 80 × 80, FOV = 210 mm, flip angle = 45°, A- > P phase encoding. Whole-brain coverage at high spatial resolution was enabled by using a multiband (MB) acceleration factor of 4, which allows to acquire 4 slices at the same time^65^. Two dummy scans were acquired prior to acquisition. During rs-fMRI acquisition, a white asterisk on a black background was displayed in the center of the stimuli screen visible inside the scanner. Participants were asked to remain awake with their eyes open and focused on the middle of the screen.

A part of our standard PET processing pipeline, a 1.5T T1-weighted MPRAGE scan was acquired for each participant with the following parameters: sagittal slice orientation, repetition time (TR) = 2110 ms, echo time (TE) = 3.58 ms, flip angle = 15°, voxel size = 1 mm isotropic. These data were collected on a Siemens Magnetom Avanto scanner at Lawrence Berkeley National Lab (LBNL). BACS protocol is to acquire a 1.5 T sMRI scan on all participants who undergo PET studies. A subset of BACS participants who undergo PET scans, including all participants in the present study, are also recruited to participate in a 3T MRI session.

### Image acquisition: PET

Detailed descriptions of FTP and PiB-PET acquisition are available in previous publications^66,67^. In the present study, only OA underwent PET scanning. All PET scans were acquired at LBNL on a Siemens Biograph 6 Truepoint PET/CT scanner in 3D acquisition mode. Prior to each PET scan a low-dose CT scan was collected for attenuation correction. FTP was synthesized at the LBNL Biomedical Isotope Facility (BIF) using a GE TracerLab FXN-Pro synthesis module with a modified protocol based on an Avid Radiopharmaceuticals protocol supplied to the facility. Participants were injected with 10 mCi of tracer and scanned in listmode 80–100 min post-injection (4 × 5 min frames). [^11^C]PiB was also synthesized at the LBNL BIF^68^. Beginning at the start of an injection of 15 mCi of PiB into an antecubital vein, 90 min of dynamic emission data were acquired and subsequently binned into 35 frames (4 × 15 s, 8 × 30 s, 9 × 60 s, 2 × 180 s, 10 × 300 s and 2 × 600 s). FTP and PiB images were reconstructed using an ordered subset expectation maximization algorithm with weighted attenuation and smoothed with a 4 mm Gaussian kernel with scatter correction (image resolution 6.5 × 6.5 × 7.25 mm^3^).

### MRI processing

sMRI and rs-fMRI data were preprocessed using Statistical Parametric Mapping software (SPM12; Wellcome Trust Centre for Neuroimaging, London, UK). sMRI data were segmented into GM, WM, and CSF compartments. Within the segmentation, native and DARTEL-imported segments were saved. Native space GM, WM and CSF segments were resliced to match the rs-fMRI resolution and used to create a GM mask ([GM > WM]*[GM > CSF]). ReHo calculations were restricted to voxels within this mask. DARTEL-imported segments from each participant were used to create a group specific template used for warping sMRI and coregistered functional and PET data to MNI space. 3T sMRI scans were also processed with the FreeSurfer (v5.3.0; http://surfer.nmr.mgh.harvard.edu/) cross-sectional pipeline to derive ROIs in each subject’s native space using the Desikan-Killiany atlas.

rs-fMRI preprocessing steps included slice timing correction, realignment to the first EPI to correct for motion and coregistration to the sMRI scan. Any participant with greater than 2 mm maximum magnitude motion was excluded. No spatial smoothing was done until after ReHo calculations^69^. Following spatial preprocessing, denoising was performed on unsmoothed data using the CONN toolbox (v18a; www.nitrc.org/projects/conn)^70^. Temporal confounding factors were regressed from each voxel BOLD timeseries and the resulting residual timeseries were bandpass filtered [0.008–0.1 Hz]. For temporal confounds we included realignment parameters, their first derivatives and the first 5 components from CSF and WM segments following an anatomical CompCor strategy in which orthogonal timeseries are estimated using principal component analysis (PCA) of the multivariate BOLD signal within each ROI^71^. We also used the artifact detection tool (ART) implemented in CONN to identify EPIs that were outliers based on motion parameters. The number of outliers varied by participant and were included as additional temporal confounds (no participant had >20% outlier volumes with a mean of 5% across all participants).

Next, spatially preprocessed, denoised images were used to calculate voxelwise ReHo (AFNI 3dReHo)^72^ within subject-specific GM masks. Because GM masks could include voxels with signal dropout in the corresponding rs-fMRI voxels, we excluded any voxels where KCC exceeded 0.49. Across both cohorts, KCC measures of 0.5 or higher were driven by functional signal dropout. For ROI analyses in native space, ReHo images were smoothed with a 6 mm^3^ kernel and then each voxelwise KCC measure was standardized by the subject-specific mean KCC across the GM. For native space regional analyses, bilateral mean normalized ReHo was calculated for each ROI in the FreeSurfer atlas. For group level standard space analyses, ReHo images were warped to MNI space using DARTEL, smoothed with 6 mm^3^ kernel, and standardized by subject-specific KCC mean in the standard cohort GM mask. This mask was created with approach analogous to the GM mask creation for each participant in native space. After segmentation into GM, WM, and CSF, the tissue compartment images for each participant were warped to MNI space using DARTEL. Once in template space, an average of each probabilistic tissue compartment was created for a total of three average images: GM, WM, and CSF. These cohort-specific averages were then used to create a binary GM mask using the same formula we applied for each participant in native space: ([GM > WM]*[GM > CSF]).

Conventional functional connectivity between the hippocampus and cortical ROIs was also calculated using CONN. To do this, we added spatial smoothing with a 6 mm^3^ kernel as part of the spatial preprocessing steps and then completed denoising as described above. We used FreeSurfer-derived ROIs and Pearson correlations between ROIs were calculated in native functional space. Pearson correlations were Fisher’s z transformed. Next, we created a functional connectivity index (FCI) measure for hippocampus. The FCI is the mean of two values: first, the average of left hippocampal connectivity with left cortical ROIs and second, the average of right hippocampal connectivity with right cortical ROIs. Average, bilateral functional connectivity of the hippocampus to AT regions (amygdala, fusiform, temporal pole, inferior temporal gyrus) and PM regions (parahippocampal cortex, retrosplenial cortex, precuneus, posterior cingulate) were also calculated.

Detrended mean hippocampal timeseries were used to calculate BOLD signal standard deviation, a measure of variability^26^. The standard deviations of left and right hippocampal timeseries were averaged to create a single hippocampal BOLD variability measure.

As part of our normal PET processing pipeline, 1.5T sMRI scans data were processed with FreeSurfer (v5.3.0) to derive native space ROIs. These ROIs were used for calculation of PiB-PET global DVR and region-specific, partial volume corrected (PVC) FTP SUVR measures (see PET image processing).

### PET image processing

DVR values for PiB-PET images were generated with Logan graphical analysis on PiB frames corresponding to 35–90 min post-injection using a cerebellar gray matter reference region^73,74^. Participants global Aβ burden was measured based on a mean cortical DVR (>1.065 used to determine PiB positivity for visualization purposes) in FreeSurfer-derived frontal, temporal, parietal and posterior cingulate ROIs^75^. Aβ in the PM network was also measured by averaging regional DVR values for the parahippocampal cortex, retrosplenial cortex, precuneus, and posterior cingulate cortex ROIs. Voxelwise PiB DVR images were warped to MNI space using SPM12, masked with a cohort-specific average intracranial mask and smoothed with a 6 mm Gaussian kernel. A mean image was created in template space to show the average tracer uptake across the cortex.

FTP standardized uptake value ratio (SUVR) images were created based on mean tracer uptake 80–100 min post-injection normalized by mean inferior cerebellar gray matter uptake^76^. FTP SUVR images were coregistered and resliced to the 1.5 T sMRI, which was closest in time to PET acquisition. Next, SUVR images were partial volume (PV) corrected using the Geometric Transfer Matrix approach^77^ on FreeSurfer-derived ROIs^27^. PV-corrected ROI SUVR values were renormalized by PV-corrected inferior cerebellar gray reference region. ERC and IT PV-corrected SUVR values were used to measure tau pathology in OA. Tau in the AT network was also calculated by averaging PV-corrected values for the amygdala, fusiform, temporal pole, and inferior temporal gyrus ROIs. Tau positivity (FTP+) was determined by measuring FTP signal in an ROI approximating Braak III/IV stages of tau pathology (SUVR > 1.26 used as threshold for positivity for visualization purposes)^6,78^. Voxelwise FTP SUVR images were warped to MNI space using SPM12, masked with a cohort-specific average intracranial mask and smoothed with a 6 mm Gaussian kernel. A mean image was created in template space to show the average tracer uptake across the cortex.

The simplified reference tissue model (SRTM) combines differential equations describing influx and efflux of a tracer from plasma into a reference region and tissue compartment^79^. After combining these differential equations, it is possible to solve for the three remaining unknown parameters using a reference region in place of arterial sampling data: the ratio of influx rate constant of target to the reference region (R1), the target efflux rate constant, and the binding potential. SRTM2 assumed a fixed value for the efflux rate constant from the reference region^80^. To examine blood flow to the hippocampus, we calculated R1 for left and right hippocampus using the cerebellum as the reference region. We averaged left and right hippocampus R1 values to derive an average hippocampal R1.

### Statistical analyses

Statistical analyses were conducted using R (https://www.R-project.org/) and jamovi (https://www.jamovi.org). A set of ANCOVA models were used to compare ReHo measures in YA and OA, adjusting for regional volume (subcortical regions) or mean thickness (cortical regions). Pearson correlations and multiple linear regression models were used to examine the relationships between demographic variables, structural measures (e.g., hippocampal volume), functional measures (e.g., ReHo, FC, variability), FTP SUVR, PiB DVR and cognition. Mediation analyses were performed using the “medmod” package in jamovi. All statistical analyses used a two-tailed level of 0.05 for defining statistical significance. Reported *p*-values were not corrected for multiple comparisons. Any exploratory analyses are explicitly described as such throughout the manuscript.

### Reporting summary

Further information on research design is available in the Nature Research Reporting Summary linked to this article.

## Supplementary information

Supplementary Information

Reporting Summary

Source Data

## Data Availability

Data used in this study will be shared by request from any qualified investigator subject to the negotiation of a data use agreement. The source data underlying Figs. 2b–d, 3a, b, e, 4a–h, 5a–d, and 6a–c, and Supplementary Figs. 1a, b, 2a–d are provided as a Source Data file.

## References

[1] Fjell AM, McEvoy L, Holland D, Dale AM, Walhovd KB (2014). What is normal in normal aging? Effects of aging, amyloid and Alzheimer’s disease on the cerebral cortex and the hippocampus. Prog. Neurobiol..

[2] Petersen RC (2000). Memory and MRI-based hippocampal volumes in aging and AD. Neurology.

[3] Frisoni GB, Fox NC, Jack CR, Scheltens P, Thompson PM (2010). The clinical use of structural MRI in Alzheimer disease. Nat. Rev. Neurol..

[4] Greicius MD, Srivastava G, Reiss AL, Menon V (2004). Default-mode network activity distinguishes Alzheimer’s disease from healthy aging: evidence from functional MRI. Proc. Natl Acad. Sci. USA..

[5] Yassa, M. A. et al. Pattern separation deficits associated with increased hippocampal CA3 and dentate gyrus activity in nondemented older adults. *Hippocampus* **21**, 968–979 (2011). 10.1002/hipo.20808 PMC3010452 20865732

[6] Braak H, Braak E (1991). Neuropathological stageing of Alzheimer-related changes. Acta Neuropathol..

[7] Hyman BT, Van Hoesen GW, Damasio AR, Barnes CL (1984). Alzheimer’s disease: cell-specific pathology isolates the hippocampal formation. Science.

[8] Maass, A., Berron, D., Libby, L. A., Ranganath, C. & Düzel, E. Functional subregions of the human entorhinal cortex. *eLife* **4**, e06426 (2015). 10.7554/eLife.06426 PMC4458841 26052749

[9] Eichenbaum H, Yonelinas AP, Ranganath C (2007). The medial temporal lobe and recognition memory. Annu. Rev. Neurosci..

[10] Bird CM, Burgess N (2008). The hippocampus and memory: insights from spatial processing. Nat. Rev. Neurosci..

[11] Ranganath C, Ritchey M (2012). Two cortical systems for memory-guided behaviour. Nat. Rev. Neurosci..

[12] Maass Anne, Berron David, Harrison Theresa M, Adams Jenna N, La Joie Renaud, Baker Suzanne, Mellinger Taylor, Bell Rachel K, Swinnerton Kaitlin, Inglis Ben, Rabinovici Gil D, Düzel Emrah, Jagust William J (2019). Alzheimer’s pathology targets distinct memory networks in the ageing brain. Brain.

[13] Harrison TM (2019). Longitudinal tau accumulation and atrophy in aging and Alzheimer’s disease. Ann. Neurol..

[14] Villain N (2012). Regional dynamics of amyloid-β deposition in healthy elderly, mild cognitive impairment and Alzheimer’s disease: a voxelwise PiB–PET longitudinal study. Brain.

[15] Braak H, Alafuzoff I, Arzberger T, Kretzschmar H, Tredici K (2006). Staging of Alzheimer disease-associated neurofibrillary pathology using paraffin sections and immunocytochemistry. Acta Neuropathol..

[16] Wu JW (2016). Neuronal activity enhances tau propagation and tau pathology in vivo. Nat. Neurosci..

[17] Yan L-R, Wu Y-B, Zeng X-H, Gao L-C (2015). Dysfunctional putamen modulation during bimanual finger-to-thumb movement in patients with Parkinson’s disease. Front. Hum. Neurosci..

[18] Kaufman SK, Del Tredici K, Thomas TL, Braak H, Diamond MI (2018). Tau seeding activity begins in the transentorhinal/entorhinal regions and anticipates phospho-tau pathology in Alzheimer’s disease and PART. Acta Neuropathol..

[19] Adams, J. N., Maass, A., Harrison, T. M., Baker, S. L. & Jagust, W. J. Cortical tau deposition follows patterns of entorhinal functional connectivity in aging. *eLife* **8**, e49132 (2019). 10.7554/eLife.49132 PMC6764824 31475904

[20] Marks SM, Lockhart SN, Baker SL, Jagust WJ (2017). Tau and β-amyloid are associated with medial temporal lobe structure, function, and memory encoding in normal aging. J. Neurosci..

[21] Sperling RA (2009). Amyloid deposition is associated with impaired default network function in older persons without dementia. Neuron.

[22] Mormino EC (2012). Aβ deposition in aging is associated with increases in brain activation during successful memory encoding. Cereb. Cortex.

[23] Huijbers W (2019). Tau accumulation in clinically normal older adults is associated with hippocampal hyperactivity. J. Neurosci..

[24] Arbabshirani MR (2014). Impact of autocorrelation on functional connectivity. Neuroimage.

[25] Zang Y, Jiang T, Lu Y, He Y, Tian L (2004). Regional homogeneity approach to fMRI data analysis. Neuroimage.

[26] Garrett DD, Kovacevic N, McIntosh AR, Grady CL (2010). Blood oxygen level-dependent signal variability is more than just noise. J. Neurosci..

[27] Baker SL, Maass A, Jagust WJ (2017). Considerations and code for partial volume correcting [18F]-AV-1451 tau PET data. Data Br..

[28] Johnson KA (2015). Tau PET imaging in aging and early Alzheimer’s disease. Ann. Neurol..

[29] Maass Anne, Lockhart Samuel N., Harrison Theresa M., Bell Rachel K., Mellinger Taylor, Swinnerton Kaitlin, Baker Suzanne L., Rabinovici Gil D., Jagust William J. (2017). Entorhinal Tau Pathology, Episodic Memory Decline, and Neurodegeneration in Aging. The Journal of Neuroscience.

[30] Lo RY (2011). Longitudinal change of biomarkers in cognitive decline. Arch. Neurol..

[31] Landau SM (2012). Amyloid deposition, hypometabolism, and longitudinal cognitive decline. Ann. Neurol..

[32] Jack CR (2011). Evidence for ordering of Alzheimer disease biomarkers. Arch. Neurol..

[33] Jack CR (2013). Tracking pathophysiological processes in Alzheimer’s disease: an updated hypothetical model of dynamic biomarkers. Lancet Neurol..

[34] Van Hoesen GW, Pandya DN, Butters N (1972). Cortical afferents to the entorhinal cortex of the Rhesus monkey. Science.

[35] Knierim JJ, Neunuebel JP, Deshmukh SS (2014). Functional correlates of the lateral and medial entorhinal cortex: objects, path integration and local-global reference frames. Phil. Trans. R. Soc. B Biol. Sci..

[36] Khan UA (2014). Molecular drivers and cortical spread of lateral entorhinal cortex dysfunction in preclinical Alzheimer’s disease. Nat. Neurosci..

[37] Olsen Rosanna K., Yeung Lok-Kin, Noly-Gandon Alix, D'Angelo Maria C., Kacollja Arber, Smith Victoria M., Ryan Jennifer D., Barense Morgan D. (2017). Human anterolateral entorhinal cortex volumes are associated with cognitive decline in aging prior to clinical diagnosis. Neurobiology of Aging.

[38] Wolk DA (2017). Medial temporal lobe subregional morphometry using high resolution MRI in Alzheimer’s disease. Neurobiol. Aging.

[39] Wilson IA, Gallagher M, Eichenbaum H, Tanila H (2006). Neurocognitive aging: prior memories hinder new hippocampal encoding. Trends Neurosci..

[40] Jacobs HIL (2018). Structural tract alterations predict downstream tau accumulation in amyloid-positive older individuals. Nat. Neurosci..

[41] Pooler AM, Phillips EC, Lau DHW, Noble W, Hanger DP (2013). Physiological release of endogenous tau is stimulated by neuronal activity. EMBO Rep..

[42] Kfoury N, Holmes BB, Jiang H, Holtzman DM, Diamond MI (2012). Trans-cellular propagation of Tau aggregation by fibrillar species. J. Biol. Chem..

[43] Busche MA (2019). Tau impairs neural circuits, dominating amyloid-β effects, in Alzheimer models in vivo. Nat. Neurosci..

[44] Jagust W (2018). Imaging the evolution and pathophysiology of Alzheimer disease. Nat. Rev. Neurosci..

[45] Kang DW (2017). Impact of amyloid burden on regional functional synchronization in the cognitively normal older adults. Sci. Rep..

[46] Bai F (2008). Default-mode network activity distinguishes amnestic type mild cognitive impairment from healthy aging: a combined structural and resting-state functional MRI study. Neurosci. Lett..

[47] Zhang Z (2012). Altered spontaneous activity in Alzheimer’s disease and mild cognitive impairment revealed by regional homogeneity. Neuroimage.

[48] Liu D (2018). Brain regional synchronous activity predicts tauopathy in 3×TgAD mice. Neurobiol. Aging.

[49] Huijbers W (2015). Amyloid-β deposition in mild cognitive impairment is associated with increased hippocampal activity, atrophy and clinical progression. Brain.

[50] Bakker A (2012). Reduction of hippocampal hyperactivity improves cognition in amnestic mild cognitive impairment. Neuron.

[51] Dickerson BC (2005). Increased hippocampal activation in mild cognitive impairment compared to normal aging and AD. Neurology.

[52] Nelson PT (2012). Correlation of Alzheimer disease neuropathologic changes with cognitive status: a review of the literature. J. Neuropathol. Exp. Neurol..

[53] Sperling RA (2018). The impact of Aβ and tau on prospective cognitive decline in older individuals. Ann. Neurol..

[54] Salami A, Wåhlin A, Kaboodvand N, Lundquist A, Nyberg L (2016). Longitudinal evidence for dissociation of anterior and posterior MTL resting-state connectivity in aging: links to perfusion and memory. Cereb. Cortex.

[55] Salami A, Pudas S, Nyberg L (2014). Elevated hippocampal resting-state connectivity underlies deficient neurocognitive function in aging. Proc. Natl Acad. Sci. USA.

[56] Sorg C (2007). Selective changes of resting-state networks in individuals at risk for Alzheimer’s disease.. Proc. Natl Acad. Sci. USA.

[57] Wang L (2006). Changes in hippocampal connectivity in the early stages of Alzheimer’s disease: evidence from resting state fMRI. Neuroimage.

[58] Palmqvist S (2017). Earliest accumulation of β-amyloid occurs within the default-mode network and concurrently affects brain connectivity. Nat. Commun..

[59] Pasquini L (2015). Link between hippocampus’ raised local and eased global intrinsic connectivity in AD. Alzheimers Dement..

[60] Das SR (2013). Increased functional connectivity within medial temporal lobe in mild cognitive impairment. Hippocampus.

[61] Tahmasian M (2015). The lower hippocampus global connectivity, the higher its local metabolism in Alzheimer disease. Neurology.

[62] Maxwell SE, Cole DA (2007). Bias in cross-sectional analyses of longitudinal mediation. Psychol. Methods.

[63] Sepulcre J (2010). The organization of local and distant functional connectivity in the human brain. PLoS. Comput. Biol..

[64] Jiang L, Zuo X-N (2016). Regional homogeneity: a multimodal, multiscale neuroimaging marker of the human connectome. Neuroscientist.

[65] Feinberg DA, Setsompop K (2013). Ultra-fast MRI of the human brain with simultaneous multi-slice imaging. J. Magn. Reson..

[66] Ossenkoppele R (2016). Tau PET patterns mirror clinical and neuroanatomical variability in Alzheimer’s disease. Brain.

[67] Schöll M (2016). PET imaging of tau deposition in the aging human brain. Neuron.

[68] Mathis CA (2003). Synthesis and evaluation of 11C-labeled 6-substituted 2-arylbenzothiazoles as amyloid imaging agents. J. Med. Chem..

[69] Zuo X-N (2013). Toward reliable characterization of functional homogeneity in the human brain: Preprocessing, scan duration, imaging resolution and computational space. Neuroimage.

[70] Whitfield-Gabrieli S, Nieto-Castanon A (2012). Conn: a functional connectivity toolbox for correlated and anticorrelated brain networks. Brain Connect..

[71] Behzadi Y, Restom K, Liau J, Liu TT (2007). A component based noise correction method (CompCor) for BOLD and perfusion based fMRI. Neuroimage.

[72] Taylor PA, Saad ZS (2013). FATCAT: (an efficient) functional and tractographic connectivity analysis toolbox. Brain Connect..

[73] Logan Jean, Fowler Joanna S., Volkow Nora D., Wang Gene-Jack, Ding Yu-Shin, Alexoff David L. (1996). Distribution Volume Ratios without Blood Sampling from Graphical Analysis of PET Data. Journal of Cerebral Blood Flow & Metabolism.

[74] Price JC (2005). Kinetic modeling of amyloid binding in humans using PET imaging and Pittsburgh Compound-B. J. Cereb. Blood. Flow. Metab..

[75] Mormino EC (2011). Relationships between β-amyloid and functional connectivity in different components of the default mode network in aging. Cereb. Cortex.

[76] Baker SL (2017). Reference tissue-based kinetic evaluation of 18F-AV-1451 for tau imaging. J. Nucl. Med..

[77] Rousset OG, Ma Y, Evans AC (1998). Correction for partial volume effects in PET: principle and validation. J. Nucl. Med..

[78] Maass A (2017). Comparison of multiple tau-PET measures as biomarkers in aging and Alzheimer’s disease. Neuroimage.

[79] Lammertsma AA, Hume SP (1996). Simplified reference tissue model for PET receptor studies. Neuroimage.

[80] Wu Y, Carson RE (2002). Noise reduction in the simplified reference tissue model for neuroreceptor functional imaging. J. Cereb. Blood. Flow. Metab..

