# Supplementary material for: Tau deposition is associated with functional isolation of the hippocampus in aging
Source: Nat Commun. 2019 Oct 25;10:4900. doi: 10.1038/s41467-019-12921-z (PMC6814780; doi:10.1038/s41467-019-12921-z)
Supplement: Supplementary file 1 — Supplementary Information [file 41467_2019_12921_MOESM1_ESM.pdf]

Tau deposition is associated with functional isolation of the hippocampus in aging

Harrison, T.M. et al.

Supplementary Notes

Potential age-related confounds of ReHo measurements

It is possible that age-related changes in cerebral flood flow drive some of the differences in ReHo we observe between YA and OA. To address this, we examined the relationship between hippocampal ReHo and hippocampal R1 derived from a simplified reference tissue model (SRTM) with dynamic amyloid PET data. R1 is the relative delivery (perfusion) parameter in the SRTM and can be used to estimate blood flow in a specific region. Within OA, there was no association between perfusion to the hippocampus and hipp-ReHo (r=-0.02, p=0.83; all r and p-value pairs reflect Pearson correlation; Supplementary Fig. 1A).

Past studies have shown that hippocampal timeseries variability changes with age.[29] We measured the standard deviation of hippocampal timeseries from each participant and showed that, as has been reported, variability was significantly lower in OA compared to YA (OA mean (SD) = 0.43 (0.10); YA = 0.66 (0.14); p<0.001; t-test). To better understand how timeseries variability is related to ReHo, we correlated hippocampal variability and hipp-ReHo in each cohort (Supplementary Fig. 2C). The relationship in YA was not statistically significant (r=-0.19, p=0.16) but was in the same direction as the borderline significant OA association (r=-0.21, p=0.047). This relationship did not survive adjustment for covariates like age, sex and hippocampal volume. Thus, hippocampal timeseries variability is a functional measure that robustly changes with age and is weakly related to hipp-ReHo such that higher ReHo corresponds to lower variability.

Having established that ReHo is associated with age, but is not driven by perfusion or timeseries variability, we wanted to better understand how hippocampal structure might influence ReHo, especially given the proximity to the inferior horn of the lateral ventricle which expands with hippocampal atrophy. We found a negative association between hipp-ReHo and hippocampal

volume (r=-0.22, p=0.03; Supplementary Fig. 1B) that did not survive adjustment for age and sex (p=0.59; multiple regression). Still, given our interest in effects beyond those that can be explained by hippocampal volume, we chose to adjust for age, sex and hippocampal volume in all our ROI analyses (raw (non-residualized) data was used for visualization).

Hipp-ReHo in YA

In YA, the relationship between hipp-ReHo and the memory domain score was not statistically significant (r=0.05, p=0.74) but ceiling effects may make a relationship difficult to observe. We also examined raw verbal episodic memory (California Verbal Learning Test Long Delay Free Recall: CVLT LDFR) scores to make it possible to combine the YA and OA cohorts. In YA, the relationship between CVLT LDFR score and hipp-ReHo was negative but not statistically significant (r=-0.15, p=0.34). As expected, across both cohorts higher hipp-ReHo predicted worse memory (r=-0.26, p=0.0003; Supplementary Fig. 2D). Notably, 14 (32%) of the 44 YA with cognitive data scored a 15 or 16 (max score 16) on the CVLT LDFR, highlighting the challenge of the ceiling effect in memory scores for YA. Finally, there was no relationship between Hipp-ReHo and working memory or executive function domain scores in YA (p's>0.479; Pearson correlation). There was also no relationship between hipp-ReHo and hipp-FC measures in YA (p's>0.16; Perason correlation; Supplementary Fig. 2A&B).

Despite robust differences between YA and OA in hippocampal timeseries variability and FC, the difference in hipp-ReHo between YA and OA was less striking. This is surprising given the association between hipp-ReHo and age within OA. One interpretation is that hipp-ReHo in YA may be a marker of risk such that individuals with higher hipp-ReHo are more vulnerable to cognitive decline when pathology-mediated disruption of hippocampal circuitry begins in older adulthood. Of course, it is possible that hipp-ReHo measured in young adulthood may not be predictive of hipp-ReHo in older age, and that higher hipp-ReHo can be driven by distinct factors

at different life phases (e.g., aging and pathology in OA). Further work is needed in young cohorts to determine the drivers of the hipp-ReHo signal in this age range and to assess the value of hipp-ReHo as a risk factor for future hippocampal circuit dysfunction.

Supplementary Figures and Legends:

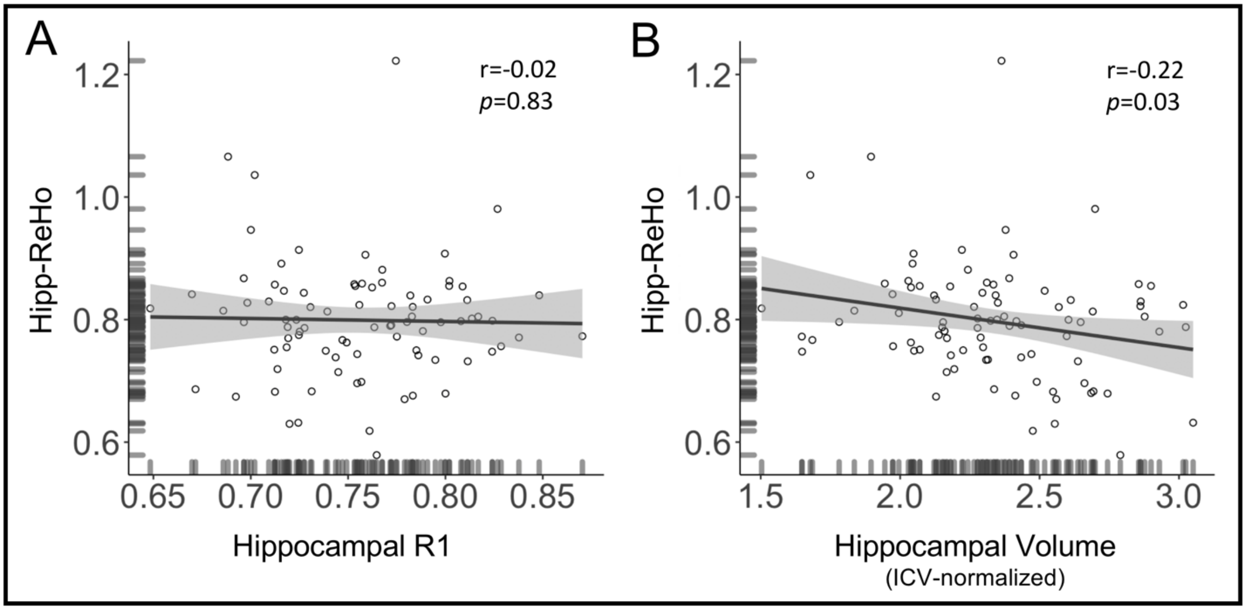

Supplementary Figure 1. The association of hipp-ReHo to perfusion and hippocampal volume. A) Hippocampal R1, a measure of perfusion to the hippocampus derived from dynamic PET data, was not a significant predictor of hipp-ReHo (r=-0.02, p=0.83; all r and p-value pairs reflect Pearson correlation). **B)** Hippocampal volume negatively predicted hipp-ReHo (r=-0.22, p=0.03). After adjusting for age and sex, hippocampal volume was no longer a significant predictor (p=0.59; multiple regression). hipp-ReHo=hippocampal regional homogeneity; ICV=intracranial volume

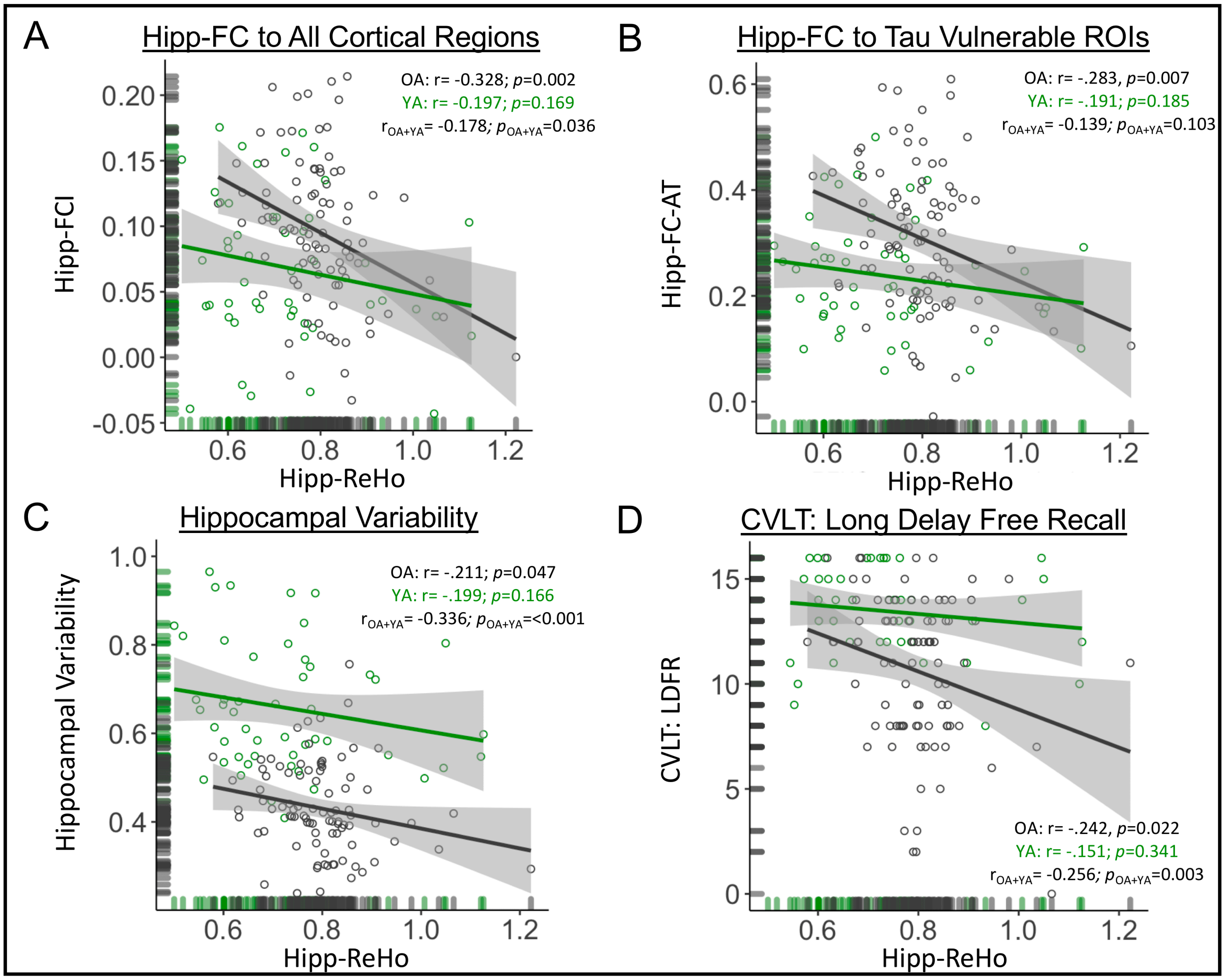

Supplementary Figure 2. Hipp-ReHo does not significantly predict functional connectivity or cognition in YA. A) Hipp-ReHo in YA (n=50; in green) was not significantly associated with hipp-FCI (r=-0.197, p=0.169; all r and p-value pairs reflect Pearson correlation), unlike in OA (n=89; in black; r=-0.328, p=0.002). Combined, there was a significant negative relationship between hipp-ReHo and hipp-FCI (r=0.-178, p=0.036). **B)** There was no association between hipp-ReHo and hipp-FC-AT (r=-0.191, p=0.185) in YA, in comparison to OA (r=-0.283, p=0.007). As a combined group, there was no significant association between hipp-ReHo and hipp-FC-AT (r=-0.139, p=0.108). **C)** There was no significant relationship between hipp-ReHo and hippocampal variability (r=-0.199, p=0.166) in YA, as opposed to OA (r=-0.211, p=0.047). After combining groups, hipp-ReHo negatively predicted hippocampal variability (r=-0.366, p<0.001). **D)** In YA (n=42), hipp-ReHo was not significantly related to CVLT LDFR (r=-0.151, p=0.341), in contrast to OA (r=-0.242, p=0.022). After combining groups, there was a significant negative relationship of hipp-ReHo to CVLT LDFR (r=-0.256, p=0.003). YA=younger adults; OA=older adults; ReHo=regional homogeneity; hipp-ReHo=hippocampal ReHo; AT=anterior temporal; FC=functional connectivity; hipp-FCI=hippocampal FC index; hipp-FC-AT=hippocampal FC to tau-vulnerable AT regions; CVLT LDFR=California Verbal Learning Test Long Delay Free Recall.

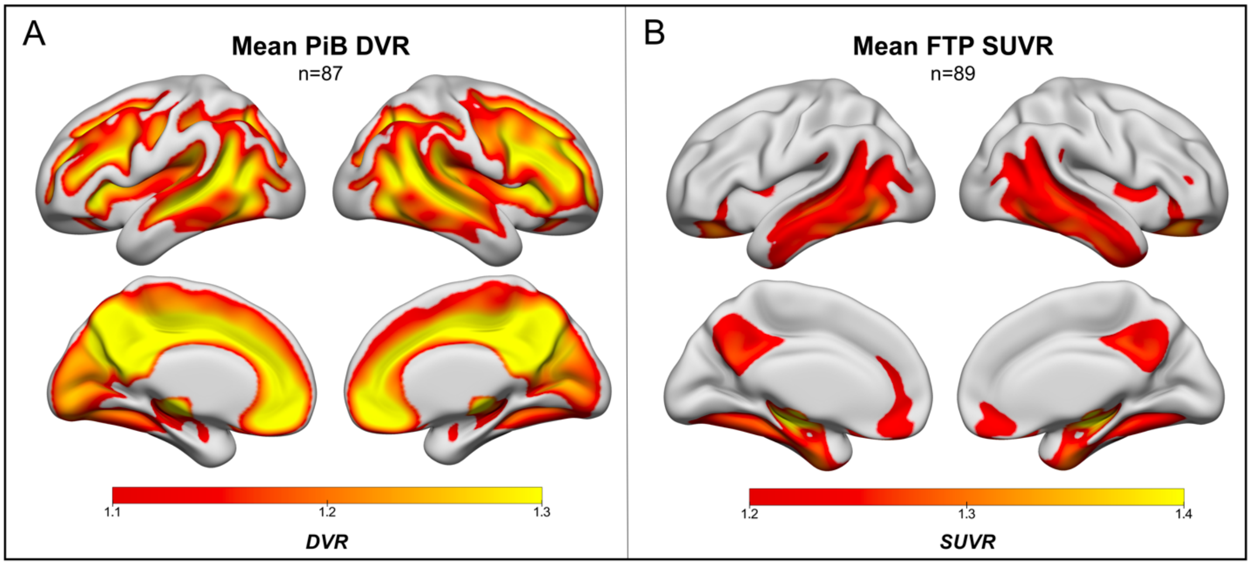

Supplementary Figure 3. Mean PiB and FTP maps show that the spatial distributions of the two tracers differ in OA. A) The average distribution of PiB tracer binding in OA (n=87) reveals uptake across much of cortex. **B)** An average FTP map for OA (n=89) reveals a temporal-predominant pattern of uptake. Uptake is also noted in posterior medial and ventromedial/orbito-frontal regions.

Supplementary Tables

Supplementary Table 1: Correlations between hippocampal ReHo and PET measures of Aβ and tau

Regional Tracer Measure	Correlation with Hippocampal ReHo	
	r	*p*
Global PiB DVR	0.29	***0.007***
PM PiB DVR	0.27	***0.011***
AT PiB DVR	0.29	***0.006***
ERC FTP SUVR	0.36	***<0.001***
PM FTP SUVR	0.15	0.156
AT FTP SUVR	0.37	***<0.001***

Bold italics indicates predictors significant at p<0.05; Pearson correlation.
PM=posteromedial; AT=anterior temporal; PiB=Pittsburgh compound B; DVR=distribution volume ratio; ERC=entorhinal cortex; FTP=flortaucipir; SUVR=standardized uptake value ratio

Supplementary Table 2: Multiple regression models predicting episodic memory performance

Model	Predictor	Estimate	SE	*t*	*p*
**1.**	Intercept	-2.2	1.9	-1.13	0.26
	Sex	-0.04	0.19	-0.24	0.81
	Age	0.02	0.02	1.13	0.26
	Hipp Vol	***1.06***	***0.32***	***3.36***	***0.001***
	Hipp-ReHo	***-2.4***	***1.03***	***-2.33***	***0.02***
				R^2=	0.21
**2.**	Intercept	-1.92	1.95	-0.99	0.33
	Sex	0.02	0.02	1.12	0.27
	Age	-0.05	0.20	-0.28	0.78
	Hipp Vol	***1.05***	***0.32***	***3.29***	***0.002***
	Hipp-ReHo	***-2.16***	***1.07***	***-2.03***	***0.046***
	PiB DVR	-0.36	0.40	-0.90	0.37
				R^2=	0.22
**3.**	Intercept	-1.08	1.97	-0.55	0.58
	Sex	-0.06	0.19	-0.33	0.74
	Age	0.02	0.02	1.08	0.28
	Hipp Vol	***0.86***	***0.33***	***2.61***	***0.011***
	Hipp-ReHo	*-1.80*	*1.06*	*-1.69*	*0.094*
	ERC FTP SUVR	*-0.77*	*0.42*	*-1.84*	*0.070*
				R^2=	0.24

***Bold italics indicates predictors significant at p<0.05*; multiple regression.** *Italics indicates predictors trending at p<0.1*. SE=standard error; Hipp Vol=hippocampal volume (normalized by intracranial volume); Hipp-ReHo=hippocampal regional homogeneity; PiB=Pittsburgh compound B; DVR=distribution volume ratio; ERC=entorhinal cortex; FTP=flortaucipir; SUVR=standardized uptake value ratio
